# Unexpected Link Between Myasthenia Gravis and Lung Adenocarcinoma: A Case Report

**DOI:** 10.1155/cris/6576971

**Published:** 2026-09-21

**Authors:** Biruke Temesgen, Fitsum A. Gemechu, Michael A. Negussie, Liya T. Tena, Mesfin Ayalew Tsegaye, Seyoum Kassa

**Affiliations:** ^1^ Department of Surgery, College of Health Sciences, Addis Ababa University, Addis Ababa, Ethiopia, aau.edu.et; ^2^ School of Medicine, College of Health Sciences, Addis Ababa University, Addis Ababa, Ethiopia, aau.edu.et; ^3^ College of Medicine and Health Sciences, Dilla University, Dilla, Ethiopia, du.edu.et

**Keywords:** case report, lung neoplasm, myasthenia gravis, paraneoplastic syndrome

## Abstract

**Introduction:**

While myasthenia gravis (MG) is commonly associated with thymic abnormalities and other autoimmune diseases, its association with malignancies, particularly lung adenocarcinoma, is exceptionally rare.

**Case Presentation:**

A 65‐year‐old male presented with bilateral eyelid drooping, double vision, and fatigable limb weakness, alongside a 4‐month history of a non‐productive cough. Neurological examination revealed bilateral ptosis, sluggish pupillary reactions, and internuclear ophthalmoplegia. Investigations demonstrated elevated anti‐acetylcholine receptor (AChR) antibody levels and nerve conduction studies (NCSs) consistent with MG. Imaging revealed a right middle lobe pulmonary nodule, confirmed as adenocarcinoma via fine‐needle aspiration. The patient showed symptomatic improvement with pyridostigmine and prednisolone and underwent surgical resection followed by chemotherapy.

**Discussion:**

Paraneoplastic neurologic syndromes (PNSs), though rare, can manifest as MG in association with malignancies. While MG is typically linked to thymoma, this case illustrates its coexistence with pulmonary adenocarcinoma, a seldom‐reported phenomenon. Cross‐reactivity between autoantibodies targeting neuronal and muscle AChRs may underlie the pathophysiology, emphasizing the need to evaluate underlying malignancies in atypical MG presentations.

**Conclusion:**

Seropositive MG can present as a paraneoplastic syndrome in association with adenocarcinoma of the lung, highlighting the importance of investigating underlying malignancies in atypical or newly diagnosed autoimmune conditions.

## 1. Introduction

Myasthenia gravis (MG) is an uncommon autoimmune disorder caused by autoantibodies targeting acetylcholine receptors (AChRs) at the neuromuscular junction, leading to impaired signal transmission [1]. It is associated with thymic abnormalities, autoimmune thyroid disorders, and systemic conditions such as rheumatoid arthritis and systemic lupus erythematosus [2]. Although MG is occasionally linked to malignancies, its association with lung cancer is very rare, particularly in cases involving AChR‐antibody (AChR‐Ab) seropositivity [3–6]. To the best of our knowledge, this is one of the few reports highlighting MG coexisting with biopsy‐confirmed adenocarcinoma of the lung.

This case has been reported in line with the SCARE criteria [7].

## 2. Case Presentation

A 65‐year‐old male presented with a 5 week history of right eyelid drooping and double vision, followed by the onset of left eyelid drooping 2 weeks later. During this period, he also experienced progressive, fatigable weakness in the right lower extremity. Additionally, he reported a 4‐month history of an intermittent, non‐productive cough.

His medical history was notable for a prior ischemic stroke on the right side with significant motor recovery. He had a smoking history of 10 pack years but quit 30 years ago. He denied any breathing difficulties, bowel or bladder incontinence.

On physical examination, his vital signs were within normal limits. Auscultation of the chest revealed clear lung fields, and the cardiovascular examination was unremarkable. Neurological examination showed bilateral ptosis, mid‐sized pupils sluggishly reactive to light, and bilateral internuclear ophthalmoplegia. Motor examination revealed normal muscle tone with 4/5 power in all extremities. Reflexes were graded 2+/4 on the right and 2/4 on the left with bilaterally down‐going plantar reflexes. No meningeal signs were noted, and the remainder of the physical examination was unremarkable.

The patient underwent several investigations. A complete blood count (CBC) revealed a hemoglobin level of 13.7 g/dL, a platelet count of 260,000/μL, and a white blood cell count of 8900/μL. Renal function tests, liver enzymes, and electrolytes were all within normal limits (Table 1). Anti‐AChR‐ab levels were elevated at 0.8 nmol/L (reference range <0.4 nmol/L).

**Table 1:** Investigation summary of the patient upon admission.

Test	Results	Reference range	Units
Hemoglobin	13.7	12.0–16.0	g/dl
MCV	92	80–100	fL
WBC	8900	4000–11,000	cells/µL
Neutrophils (%)	78.4%	40%–75%	%
AST	15	0–32	U/L
ALT	27	0–35	U/L
ALP	62	35–104	U/L
Total bilirubin	0.8	0.1–1.2	mg/dl
Direct bilirubin	0.2	0.0–0.3	mg/dl
Albumin	2.7	3.5–5.0	g/dl
PT	13.7	11.4–17.7	Seconds
aPTT	27.1	24.2–36.3	Seconds
INR	1.16	1.0	Ratio
Anti‐AchR‐Ab	0.8	<0.4	Nmol/Lit

A chest X‐ray demonstrated a well‐circumscribed nodule in the right middle lobe, measuring ~2 cm in diameter, without calcification or hilar lymphadenopathy. Brain MRI revealed bilateral inferomedial thalamic gliosis, likely a sequela of chronic Percheron infarction, with no evidence of acute ischemia or hemorrhage.

**Figure 1:**
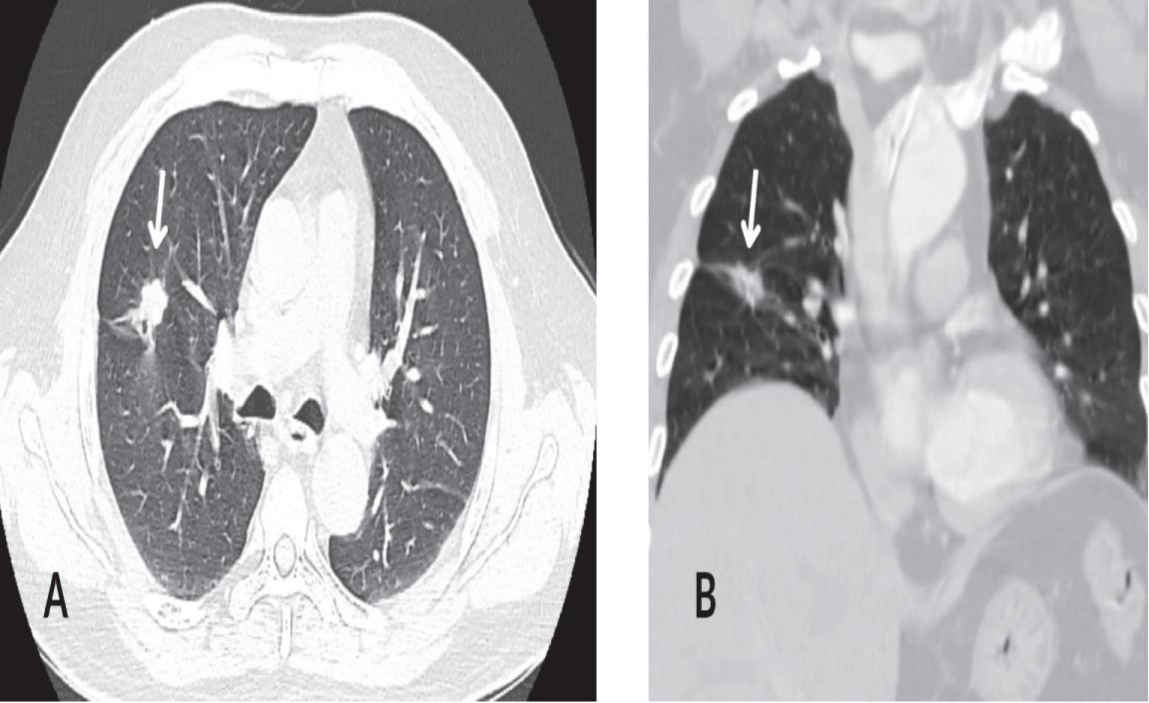
Axial (A) and coronal (B) CT images of the chest demonstrate a 2.1 cm spiculated pulmonary nodule located in the lateral segment (S4) of the right middle lobe (arrow). The lesion exhibits irregular margins with radiating spicules, raising suspicion for a malignant etiology.

Chest CT with contrast confirmed the presence of a lobulated, spiculated, and irregular nodule in the lateral segment of the right middle lobe, measuring 2.1 cm in diameter, with no abnormal lymphadenopathy (Figure 1). CT‐guided fine‐needle aspiration cytology (FNAC) of the mass revealed a few crowded clusters of moderately pleomorphic polygonal cells with a high nuclear‐to‐cytoplasmic ratio, hyperchromasia, conspicuous nucleoli, and frequent foamy macrophages, consistent with malignancy.

Nerve conduction studies (NCSs) revealed a significant decremental response of 20% in the compound muscle action potential (CMAP) amplitude during repetitive nerve stimulation at 3 Hz, consistent with a neuromuscular junction disorder. Sensory nerve action potentials (SNAPs) were preserved, and motor conduction velocities were within normal limits, further supporting a diagnosis of MG.

The patient was initiated on pyridostigmine and prednisolone, resulting in symptomatic improvement. After 1 month of treatment, he underwent right posterolateral thoracotomy and right pneumonectomy under general anesthesia. Intraoperative findings were a 4 cm × 4 cm hard nodular mass at the confluence of the oblique and horizontal fissures, causing scarring of all three lobes. Multiple peribronchial lymphadenopathies were noted. Although right pneumonectomy is associated with increased morbidity and is generally avoided when lung‐sparing resection is feasible, it was deemed necessary in this case due to intraoperative findings. The tumor was located at the confluence of the oblique and horizontal fissures, with dense scarring involving all three lobes, precluding safe anatomical separation. As a result, complete resection with negative margins could not be achieved with lobectomy or bilobectomy, and pneumonectomy was required to ensure adequate oncologic clearance. No anesthesia‐related complications occurred. Pathological examination of the excised tissue confirmed adenocarcinoma. Microscopically, invasive glandular structures were observed infiltrating the stroma, lined by atypical columnar cells with hyperchromatic nuclei, prominent nucleoli, and moderate eosinophilic cytoplasm. Mitotic activity was evident, and desmoplastic stromal reactions were present. Immunohistochemical analysis of the tumor demonstrated positive expression of AChR‐related antigens, supporting a potential paraneoplastic mechanism linking the malignancy to the patient’s MG. Peribronchial lymph nodes showed reactive hyperplasia without metastatic involvement. Based on the postoperative pathological findings, the tumor was staged as pT2aN0M0, corresponding to Stage IB disease according to the TNM classification. The patient was subsequently referred to the oncology department for adjuvant chemotherapy. During 3 months of follow‐up in the outpatient clinic, no new complaints were reported. Notably, during the follow‐up period after tumor resection, the patient demonstrated marked improvement in neuromuscular symptoms, providing further clinical support for a paraneoplastic association between lung adenocarcinoma and MG.

## 3. Discussion

Paraneoplastic neurologic syndromes (PNSs) are rare, occurring in fewer than 1% of cancer patients, and result from indirect effects of primary tumors. They are often seen in lung, ovarian, and breast cancers [8]. In lung cancer, small‐cell lung cancer (SCLC), comprising about 15% of cases, is frequently linked to PNS, such as Lambert–Eaton myasthenic syndrome (LEMS) [9, 10].

Adenocarcinoma, the most common lung cancer type, accounts for over 40% of cases [11, 12]. Paraneoplastic syndromes are less common in adenocarcinoma compared to SCLC. Hypertrophic pulmonary osteoarthropathy (HPO) is the most frequent paraneoplastic syndrome linked to adenocarcinoma, occurring in 0.72% of lung cancer patients [8, 13]. Other rarer paraneoplastic manifestations include dermatomyositis/polymyositis and Trousseau’s syndrome [8]. This case of adenocarcinoma with seropositive MG represents an exceedingly rare paraneoplastic manifestation. While PNSs are well described in lung cancer, particularly in association with SCLC, their occurrence in lung adenocarcinoma remains uncommon [8‐Seropositive10]. MG, in contrast to LEMS, is rarely associated with primary lung malignancies and is more frequently linked to thymic pathology or other autoimmune conditions [2, 14, 15]. In this patient, several findings support a possible paraneoplastic association. The patient demonstrated elevated anti‐AChR‐ab levels, and immunohistochemical analysis of the tumor revealed expression of AChR‐related antigens, suggesting a potential mechanism of ectopic antigen expression and immune cross‐reactivity. Autoantibodies, specifically anti‐AChR or anti‐MuSK, target nicotinic AChRs (nAChRs). These receptors include the α1, α3, and α7 subunits, with the α3 subunit found in the thymus and hypothesized to contribute to the pathogenesis of MG [16]. In 1992, Chini et al. [17] demonstrated that SCLC, non‐small‐cell lung carcinoma, and neuroblastomas have the potential to express the α3 subunit of nAChRs. They further established the cross‐reactivity of autoantibodies against α3‐nAChRs with α1‐nAChRs [17–19].

The diagnosis of MG typically relies on the detection of anti‐AChR or anti‐MuSK antibodies. Alternatively, electrophysiological testing through repetitive nerve stimulation can be employed to identify neuromuscular transmission defects. Diagnosis is confirmed by serological evidence of anti‐AChR antibodies, along with supportive findings from electromyography (EMG) and NCSs [17].

Managing MG as a paraneoplastic syndrome requires treating the malignancy and the autoimmune response [20, 21]. Acute exacerbations are controlled with immunomodulatory therapies like corticosteroids, IVIG, or plasma exchange. Long‐term symptom control often involves immunosuppressive agents such as azathioprine or mycophenolate mofetil [20]. Advanced treatments, such as eculizumab, are reserved for refractory cases [22]. This patient was treated with surgical resection of the lung mass, acetylcholinesterase inhibitors, and low‐dose steroids, achieving symptom control. Following surgery, the patient was referred for chemotherapy.

## 4. Conclusion

Seropositive MG can present as a paraneoplastic syndrome in association with adenocarcinoma of the lung, highlighting the importance of investigating underlying malignancies in atypical or newly diagnosed autoimmune conditions. This case report was prepared in accordance with the CARE guidelines. The completed CARE Checklist is available in the Supporting Information.

## Funding

No funding was received for this manuscript.

## Disclosure

A limitation of this case report is the unavailability of the original immunohistochemical images for inclusion in the manuscript, which limits independent visual verification of the reported immunohistochemical findings. All authors contributed to this research and read and approved the final version of the manuscript.

## Consent

Institutional ethics committee/Institutional Review Board (IRB) approval was not required for this single‐patient case report because it did not constitute human‐subjects research under institutional policy. Written informed consent was obtained from the patient for the publication of the clinical details, images, and related discussion. A copy of the written consent is available for review by the Editor‐in‐Chief upon request.

## Conflicts of Interest

The authors declare no conflicts of interest.

## Supporting Information

Additional supporting information can be found online in the Supporting Information section.

## Supporting information


**Supporting Information** CARE Checklist. The completed CARE Checklist is available in the Supporting Information.

## Data Availability

The data supporting the findings of the case are available upon request to the corresponding author.
